# Efficacy of a Modified Hysteroscopic Proximal Tubal Occlusion Technique on IVF Outcomes

**DOI:** 10.3389/fmed.2025.1609296

**Published:** 2025-10-28

**Authors:** Tao-Tao Hu, Li Sun, Kai Yuan, Wen-Yu Liu, Yi-ling Cai, Hua-Lei Cai

**Affiliations:** 1 Department of Obstetrics and Gynecology, Guizhou Medical University, Guiyang, China; 2 Department of Gynecology, Bai Yun Hospital Affiliated to Guizhou Medical University, Guiyang, China; 3 Department of Obstetrics and Gynecology, Affiliated Hospital of Guizhou Medical University, Guiyang, China

**Keywords:** hysteroscopy, *in vitro* fertilization-embryo transfer (IVF-ET), microcoil, tubal hydrosalpinx, tubal embolization

## Abstract

**Objective:**

This study aimed to evaluate whether optimizing the placement of microcoil in the fallopian tube during hysteroscopy could enhance *in vitro* fertilization-embryo transfer (IVF-ET) pregnancy rates and reduce complications.

**Methods:**

A retrospective cohort study included 94 patients with tubal infertility who underwent tubal embolization at Baiyun Hospital of Guizhou Medical University between May 2019 and December 2023. Patients were divided into two groups: Group A (*n* = 65) used the original technique, while Group B (*n* = 29) used a modified technique. Effective follow-up data were obtained from 42 patients in Group A (Group Aa) and 15 patients in Group B (Group Ba). Key variables, including the mean time to successful IVF pregnancy, the number of IVF cycles, ongoing pregnancy (gestation ≥12 weeks), and complication rates, were analyzed.

**Results:**

The mean time to conception was shorter in Group Ba (6.10 months, median = 7.5 months) than in Group Aa (11.58 months, median = 12 months), though not statistically significant (*p* = 0.092). Similarly, the mean number of IVF cycles required for implantation was slightly lower in Group Ba (1.60) than in Group Aa (1.92), with no significant difference (*p* = 0.236). However, clinical pregnancy persistence rates were significantly higher in Group Ba (10/14, 71.43%) than in Group Aa (12/32, 37.50%) (*χ*^2^ = 4.493, *p* = 0.034). Additionally, Group Aa reported two cases of post-IVF ectopic pregnancy (2/32), while no such cases were observed in Group Ba.

**Conclusion:**

Optimizing the proximal tubal plugging site via hysteroscopy may enhance ongoing pregnancy post-IVF-ET. Further studies are needed to explore the relationship between coil positioning depth and ectopic pregnancy risk, as well as to determine the optimal timing for postoperative IVF-ET. Ongoing pregnancy rates differed markedly by occlusion site: unilateral isthmic occlusion (0%) vs. bilateral isthmic occlusion (44.44%, *p* = 0.059), suggesting a clinical trend. Conversely, interstitial occlusion yielded high and comparable rates (unilateral: 83.33% vs. bilateral: 62.50%, *p* = 0.393), unaffected by laterality.

## 1 Introduction

Tubal hydrosalpinx has been demonstrated to significantly reduce implantation and pregnancy rates, leading to high IVF failure rates in affected patients (1). The mechanisms include embryo flushing or reflux caused by tubal fluid, direct toxic effects of hydrosalpinx fluid on embryos, and altered expression of key endometrial factors such as HOX10, which diminishes endometrial tolerance and pregnancy success.

Currently, salpingectomy is the most common clinical treatment for hydrosalpinx (2). Although studies have shown that this approach improves postoperative IVF-ET outcomes in patients with hydrosalpinx (3, 4), it is an invasive transabdominal procedure associated with risks such as visceral injury, vascular damage, and pelvic adhesions (5). Additionally, some research studies indicate that tubal resection may reduce ovarian reserve by affecting ovarian blood flow (6) or ovarian responsiveness (7).

To address these limitations, less invasive alternatives such as laparoscopic proximal tubal occlusion, tubal ligation, and hysteroscopic insertion of the Essure intra-tubal device have been explored. These methods aim to preserve ovarian function while minimizing procedural trauma, thereby improving post-IVF-ET pregnancy outcomes (3, 8–11).

At our center, transhysteroscopic microcoil embolization has been used to optimize perioperative indicators and health economic benefits, achieving surgical success rates comparable to those of laparoscopic procedures through dual mechanisms of physical blockage and microenvironmental modulation (12). However, clinical observations revealed a case of tubal interstitial pregnancy following bilateral embolization, prompting further investigation into its causes. Two primary factors were identified: (i) the inherent risk of tubal pregnancy associated with the IVF-ET technique itself and (ii) the excessive depth of microcoil placement (1–2 cm into the tubal lumen via the uterine horn catheter, equivalent to the isthmic portion), which left a proximal residual lumen and increased the risk of ectopic pregnancy.

To mitigate this risk, we refined the technique by adjusting the microcoil placement to <1 cm into the tubal lumen, corresponding to the interstitial portion. This study prospectively compared the IVF-ET outcomes between patients undergoing isthmic embolization (Group A) and those undergoing interstitial tubal embolization (Group B). By analyzing postoperative IVF cycles, implantation rates, pregnancy sustainability, time to IVF, and complication rates, this study aimed to provide evidence-based insights for optimizing pretreatment strategies in patients with tubal hydrosalpinx.

## 2 Methods

### 2.1 Study design and population

This study included 94 infertile patients with unilateral or bilateral hydrosalpinx confirmed by hysterosalpingography or vaginal ultrasound. The inclusion criteria were as follows: ① individuals with no systemic diseases such as coagulation disorders; ② women of childbearing age with reproductive requirements; ③ patients diagnosed with unilateral or bilateral hydrosalpinx via hysterosalpingography, vaginal ultrasound, or laparoscopy before surgery; and ④ individuals with no genital deformities or contraindications to hysterolaparoscopic surgery. The exclusion criteria were as follows: ① patients having a combination of serious organic diseases, contraindications to hysteroscopic surgery, and anesthesia allergy and ② those with excessive endosalpinx hyperplasia or obvious inflammation, congestion, and edema of the endosalpinx that prevented the tubal opening from being exposed on days 1–3 of the menstrual cycle.

### 2.2 Surgical procedures

Group A (65 patients before the modified technique) underwent surgery within 1–3 days after the end of menstruation. Preoperative preparations included detailed doctor–patient communication, during which the advantages and disadvantages of tubal embolization under hysteroscopy, as well as intraoperative and postoperative risks, were explained to the patients. Patients voluntarily chose to undergo the appropriate surgical treatment and signed the surgical agreement. Routine preoperative examinations were completed to exclude contraindications to surgery.

**Figure 1:**
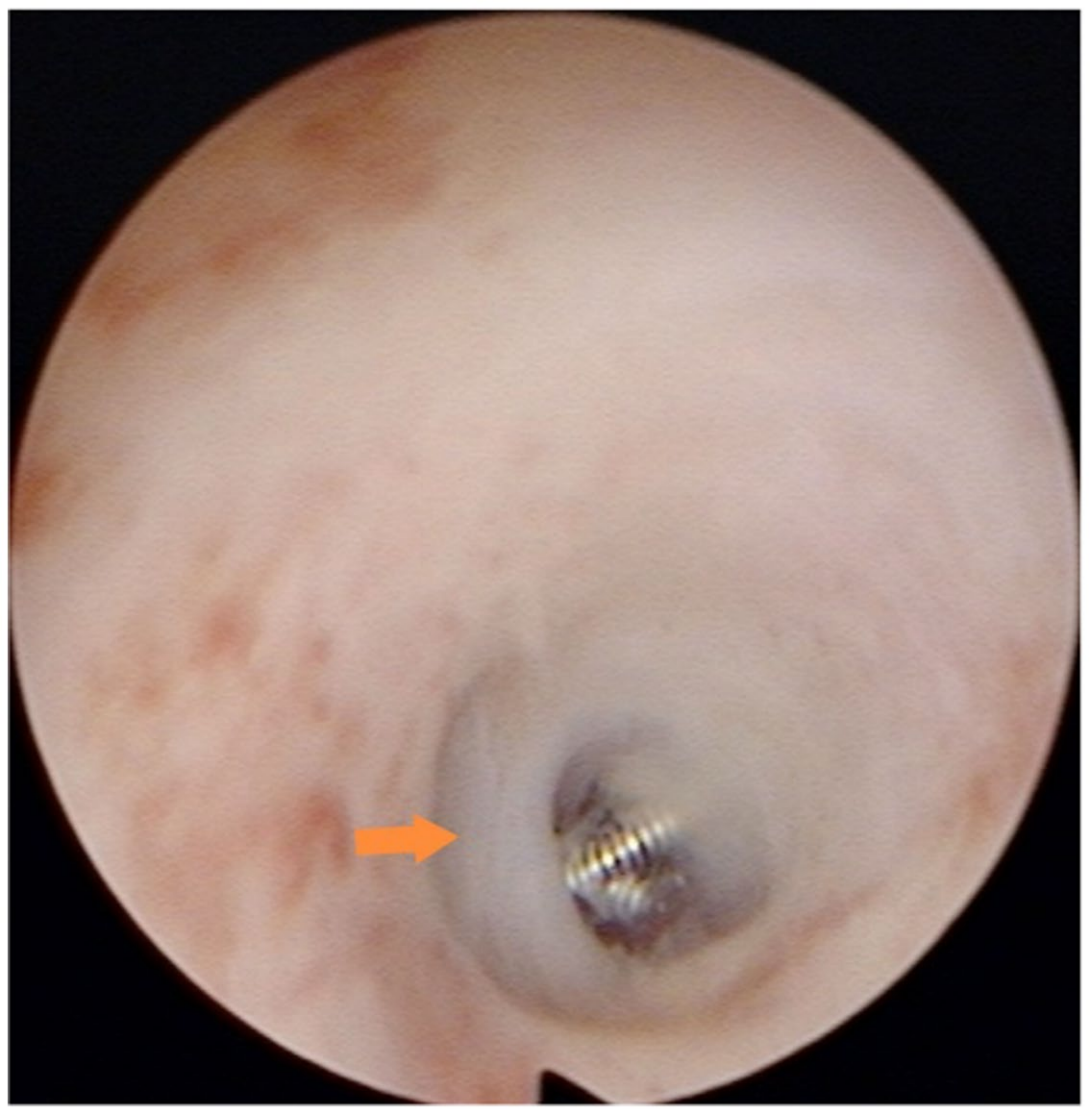
Microcoil placement in the isthmus: the microcoil was deployed in the isthmic portion of the fallopian tube, approximately 1–2 cm from the tubal ostium. Consequently, the hysteroscopic view revealed only the distal end of the microcoil protruding slightly from the ostium.

Preoperative medication included clindamycin hydrochloride dextrose injection administered 30 min prior to surgery to prevent possible infection. The specific surgical steps were as follows: The patient was required to empty the bladder and assume the bladder lithotomy position. Routine sterilization was performed. After sterilization, the cervical area was exposed, and an anesthetic consisting of 5 mL of 0.75% levobupivacaine hydrochloride injection diluted with 0.9% sodium chloride injection was injected into the cervix at the 5 o’clock and 7 o’clock positions. A probe was used to determine the depth and direction of the uterine cavity, followed by gradual widening of the cervical opening to size 6.5–7 using a uterine dilatation wand. The uterine distension pressure was set in the range of 100–120 mmHg, the contents of the irrigation tube were removed, and the hysteroscope was inserted to complete the uterine examination. A uterine horn catheter was inserted at the opening of the fallopian tube, through which the inner catheter was fed into the lumen of the tube for approximately 1–2 cm and the microcoil was placed (Figure 1). The operation was considered complete if the tail filament could be observed at the uterine horn. Patients were required to return to the hospital for ultrasound review at 1 and 3 months after the operation.

**Figure 2:**
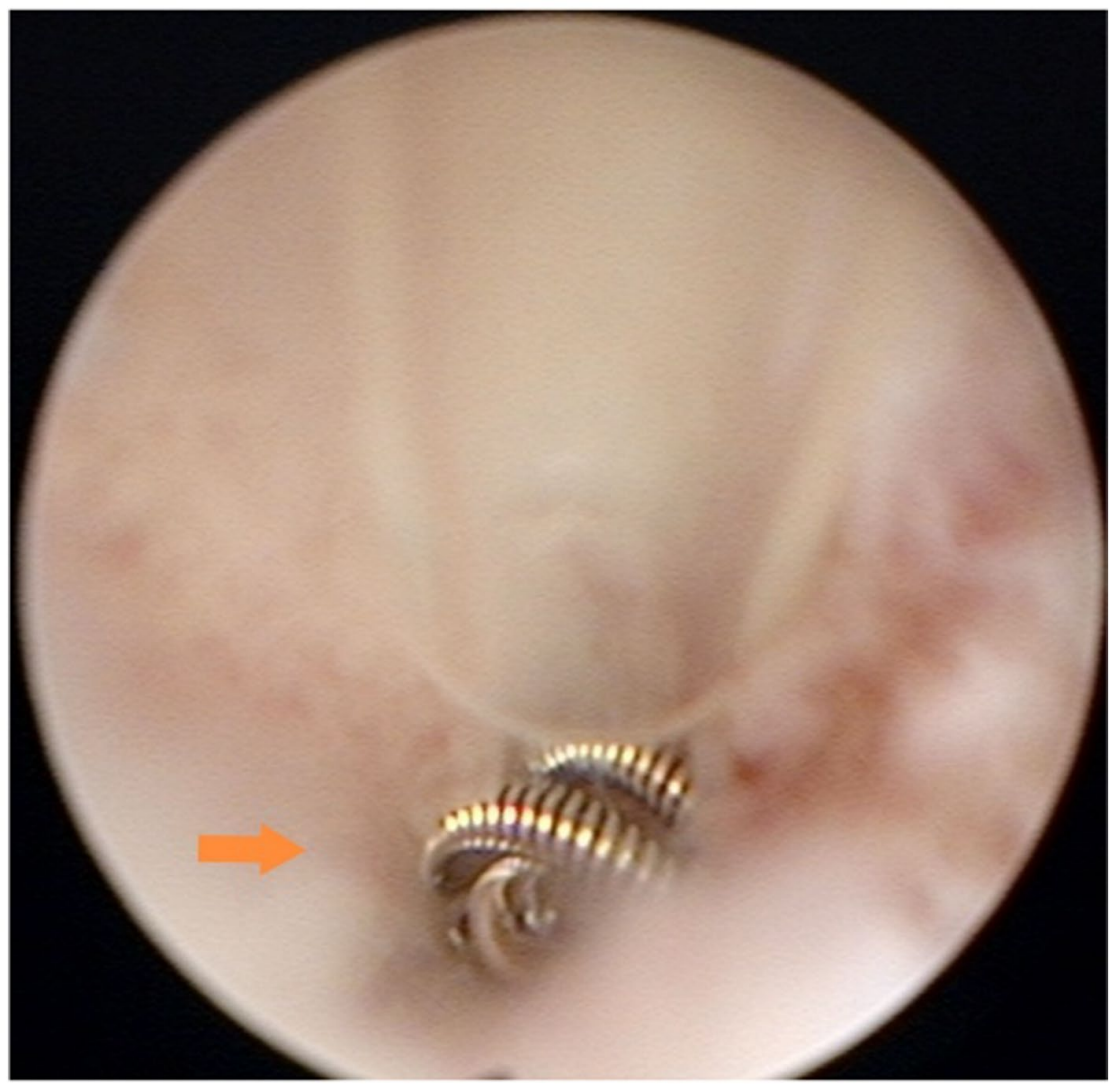
Microcoil placement in the interstitium: the microcoil was deployed within the interstitial portion of the fallopian tube (0.5–1 cm from the ostium). Under hysteroscopy, approximately three loops of the microcoil were visible, concentrically stacked at the tubal ostium.

Group B (29 patients after the modified technique) underwent surgical timing, preoperative preparation, and surgical methods that were identical to those of Group A for the initial steps. However, in step 6, the uterine horn catheter was placed at the opening of the fallopian tube, and the inner catheter was placed through the uterine horn catheter into the lumen of the fallopian tube to a depth of less than 1 cm for the placement of the spring coil. This adjustment was made to reduce the risk of tubal interstitial pregnancy by minimizing the depth of coil placement (Figure 2).

### 2.3 Observational indices

After 1–3 years of follow-up, embryo transfer outcomes and IVF cycle data were analyzed in effective respondents (Group Aa = 42, Group Ba = 15). Outcomes included ongoing pregnancies (≥12 weeks), implantation failure, and complications.

**Figure 3:**
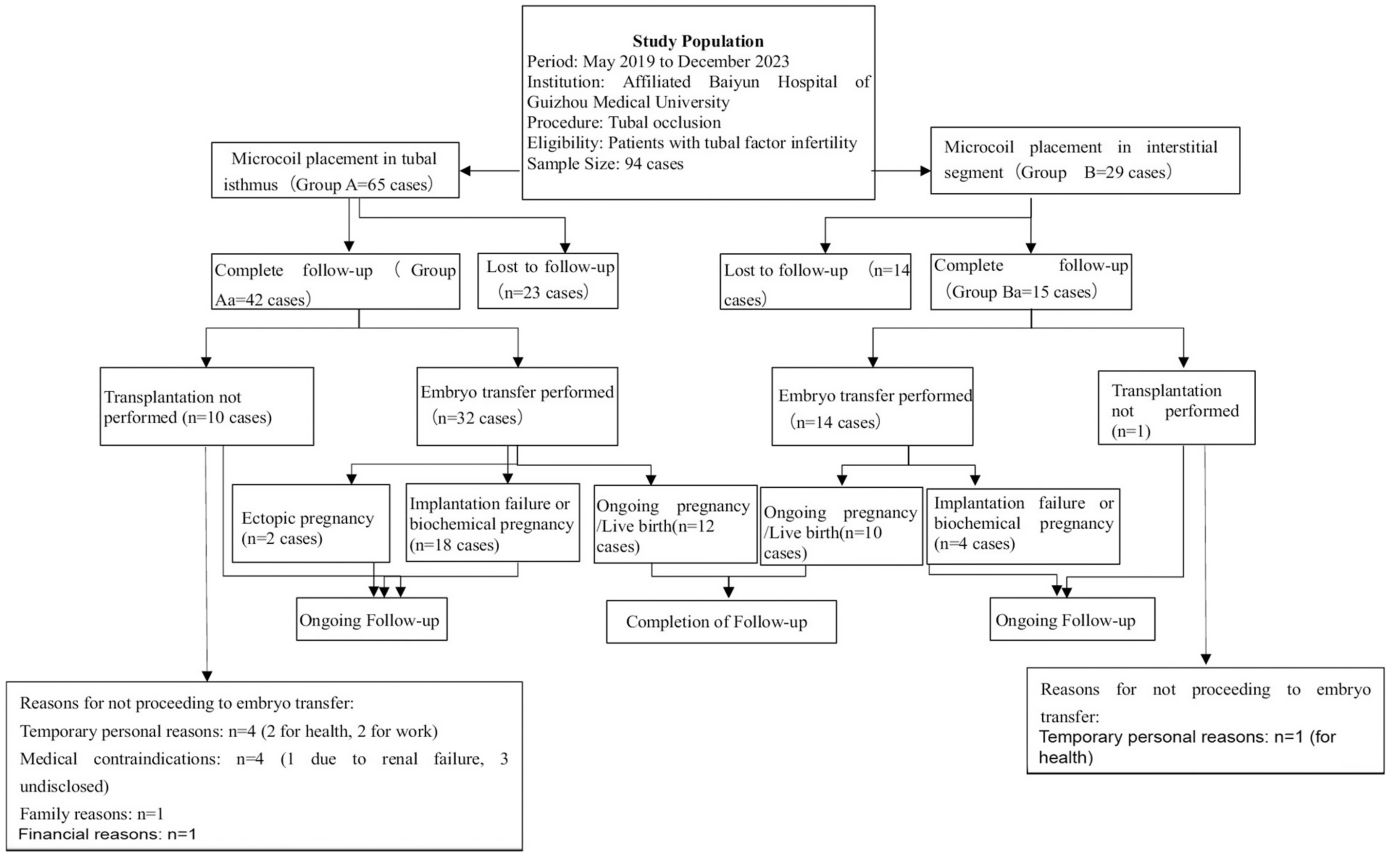
Flowchart of patient selection, tubal occlusion procedure, and follow-up outcomes in the study on tubal factor infertility.

Patients were followed up for 1–3 years postoperatively. Excluding patients lost to follow-up, the number of recipients who underwent transplantation following the procedure was documented, along with those who did not. Pregnancy outcomes after transplantation were evaluated, including the number of ongoing clinical pregnancies (defined as gestation lasting ≥12 weeks), instances of implantation failure or biochemical pregnancy, and postoperative complications. Effective follow-up responders were divided into Group Aa (*n* = 42) and Group Ba (*n* = 15). Data collected for analysis included the number of postoperative IVF cycles, implantation status, pregnancy sustainability, interval to IVF, and complications (see Figure 3).

### 2.4 Statistical analysis

All statistical analyses were performed using SPSS version 27. Continuous data are presented as mean ± SD, and categorical variables are presented as frequencies and percentages. Comparisons used the chi-squared tests for categorical data and independent *t*-tests for normally distributed continuous data. A two-tailed *p*-value of < 0.05 was considered statistically significant.

## 3 Results

### 3.1 Follow-up results

A total of 94 patients with unilateral or bilateral hydrosalpinx underwent embolization and were followed up for 1–3 years postoperatively. Of these, 57 patients responded to the follow-up. In Group A (65 patients), 42 responded to the follow-up. Among them, 32 underwent transplantation [12 clinically sustained pregnancies, 18 unsuccessful pregnancies (not implanted/biochemical), and 2 ectopic pregnancies], while 10 patients did not undergo transplantation due to physical factors or family financial reasons. In Group B (29 patients), 15 responded to the follow-up. Among them, 14 underwent transplantation [10 clinically sustained pregnancies and 4 unsuccessful pregnancies (not implanted/biochemical)], while 1 patient did not undergo transplantation due to physical factors or family financial reasons.

### 3.2 Baseline data comparison

After excluding patients lost to follow-up, preoperative baseline data of the 57 follow-up responders were analyzed. No significant differences were observed between the two groups in terms of age, number of pregnancies, number of births, years of infertility, and number of preoperative IVF cycles (Table 1).

**Table 1:** Baseline characteristic comparison between the groups (mean ± SD).

Characteristics	Group Aa (*n* = 42)	Group Ba (*n* = 15)	*t*	*p*
Age (years)	34.55 ± 4.82	34.67 ± 4.40	−0.084	0.933
Gravidity	1.17 ± 1.17	1.40 ± 1.18	−0.662	0.510
Parity	0.29 ± 0.46	0.27 ± 0.46	0.138	0.890
Duration of infertility	6.19 ± 4.40	4.87 ± 5.04	0.963	0.340
Number of preoperative IVF cycles	1.17 ± 1.34	0.67 ± 0.98	1.320	0.192

These data were analyzed using independent samples *t*-tests. Measurement data are expressed as mean ± SD.

### 3.3 Time to conception and IVF cycles

Group Ba demonstrated a numerically shorter time to conception than Group Aa (6.10 months vs. 11.58 months; *p* = 0.092). A similar trend was observed in the mean number of postoperative IVF cycles (1.60 vs. 1.92; *p* = 0.236). Although these differences were not statistically significant, they suggest a potential clinical advantage for the modified technique that warrants further investigation into larger studies (see Table 2).

**Table 2:** Comparison of time to conception and IVF cycles in patients with ongoing pregnancy between the groups (mean ± SD).

Characteristics	Gestation ≥12 weeks in group Aa (*n* = 12)	Gestation ≥12 weeks in group Ba (*n* = 10)	*t*	*p*
Number of postoperative embryo transfer cycles	1.92 ± 0.67	1.60 ± 0.52	1.223	0.236
Time intervals from surgery to conception	11.58 ± 9.19	6.10 ± 3.63	1.769	0.092

### 3.4 Clinical pregnancy outcomes

A significant difference was observed between Group Aa and Group Ba in terms of clinically sustained pregnancies and unsuccessful pregnancies (not implanted/biochemical). Specifically, the clinical pregnancy rate in Group Ba was 71.43%, which was significantly higher than the 37.50% in Group Aa (*χ*^2^ = 4.493, *p* = 0.034) (Table 3). Conversely, the rate of unsuccessful pregnancies (not implanted/biochemical) was 62.50% in Group Aa, which was significantly higher than the 28.57% in Group Ba. These findings indicate that the placement of a microcoil into the interstitium (Group Ba) resulted in superior clinical outcomes compared to placement in the isthmus (Group Aa).

**Table 3:** Comparison of clinical pregnancy outcomes between the groups *n* (%).

Characteristics	Microcoil placement in the isthmic segment (Aa = 32)	Microcoil placement in the interstitial segment (Ba = 14)	Total	*χ* ^2^	*p*
Number of ongoing pregnancies/live births	12 (37.50)	10 (71.43)	22 (47.83)	4.493	0.034
Number of failure of ongoing pregnancy	20 (62.50)	4 (28.57)	24 (52.17)		

Compare the difference in frequency distributions using the chi-squared test.

### 3.5 Subgroup analysis: impact of occlusion laterality by tubal segment

A subgroup analysis was conducted to assess the potential influence of occlusion laterality (unilateral vs. bilateral) on IVF outcomes, stratified by the anatomical site of microcoil placement (isthmic vs. interstitial).

As detailed in Table 4, marked differences in ongoing pregnancy rates were observed within the isthmic occlusion cohort. The unilateral occlusion group demonstrated an ongoing pregnancy rate of 0%, contrasting with 44.44% in the bilateral occlusion group. Although this considerable numerical difference did not reach formal statistical significance (*p* = 0.059), it suggests a potential clinical trend warranting consideration. Conversely, within the interstitial occlusion cohort, ongoing pregnancy rates were comparable between the unilateral (83.33%) and bilateral (62.50%) groups, confirming no statistically significant association (*p* = 0.393).

**Table 4:** Comparison of IVF pregnancy outcomes between unilateral and bilateral tubal occlusion at different coil placement sites.

Occlusion site	Laterality	Number of patients	Ongoing pregnancies, *n* (%)	*χ* ^2^	*p*
Isthmic	Unilateral	5	0 (0)	3.556	0.059
	Bilateral	27	12 (44.44)		
Interstitial	Unilateral	6	5 (83.33)	0.729	0.393
	Bilateral	8	5 (62.50)		

Compare the difference in frequency distributions using the chi-squared test.

## 4 Discussion

This study compared two microcoil placement techniques in patients with hydrosalpinx undergoing *in vitro* fertilization and embryo transfer (IVF-ET). Although the differences did not reach statistical significance, the modified technique (interstitial placement) demonstrated a reduction in the time to conception and the number of required IVF cycles. Notably, the clinical pregnancy rate was significantly higher in the modified technique group, while the rate of unsuccessful pregnancies (including implantation failure and biochemical pregnancy) was lower.

Anatomically, the interstitial portion of the fallopian tube, being the narrowest segment with a diameter of approximately 0.5–1 mm and a length of approximately 1 cm, offers a smaller luminal space compared to the isthmus (diameter 1–2 mm, length 2–3 cm). This anatomical characteristic enables microcoils placed in the interstitium to achieve tighter apposition to the tubal wall, thereby reducing the risk of displacement or detachment. The constrained luminal environment facilitates sufficient contact between the nylon threads and the tubal endometrium, promoting adhesion formation and more effectively preventing the reflux of hydrosalpinx fluid into the uterine cavity.

During clinical follow-up, two cases of microcoil migration were observed, both occurring toward the ampullary portion of the fallopian tube, while no microcoil detachment was noted in the interstitial segment. These findings further support the superior mechanical stability and occlusion efficacy of interstitial placement over isthmic placement, which was consistent with the anatomical advantages described. The ampulla, as the widest and most dynamically active segment of the fallopian tube, possesses a relatively spacious lumen that may diminish microcoil–wall contact. Moreover, its complex mucosal folds may compromise optimal microcoil anchoring, increasing the risk of migration under tubal peristalsis or fluid dynamics.

Regarding the potential impact of microcoil migration, microcoils retained within the ampulla may cause localized mechanical irritation to the tubal mucosa. However, given the distance from the uterine cavity, the direct effect on endometrial receptivity is likely limited. In contrast, complete microcoil detachment leading to hydrosalpinx recurrence or fluid reflux may allow inflammatory mediators to enter the uterine cavity, potentially impairing embryo implantation. It should be noted that the number of migration events in this study was small, and no significant adverse pregnancy outcomes were observed. Nevertheless, the long-term implications warrant further evaluation in larger cohorts.

Both open and laparoscopic tubal ligation or salpingectomy preserve the interstitial portion of the fallopian tube, which remains a potential site for interstitial pregnancy following IVF-ET. Such pregnancies account for approximately 0.8% of all ectopic pregnancies after IVF-ET (13). When microcoils are deployed in the isthmic portion, the persistence of a residual lumen proximal to the microcoil within the interstitial segment may allow embryo migration and implantation, resulting in interstitial pregnancy. By advancing the occlusion site to the interstitial segment, we observed not only improved rates of ongoing pregnancy, reduced medical costs, and fewer treatment cycles but also an absence of further microcoil migration or interstitial pregnancy.

Our findings also indicate that the laterality of tubal occlusion does not significantly influence IVF outcomes, particularly following interstitial microcoil placement. This finding supports the premise that the efficacy of the procedure stems primarily from the mechanical isolation of hydrosalpinx fluid rather than the number of tubes occluded. These results justify targeted unilateral occlusion in cases of unilateral hydrosalpinx, preserving the functional integrity of the contralateral tube without compromising pregnancy rates.

The transcervical microcoil embolization technique used in this study shares a similar mechanism of action to that of the internationally reported Essure system—both achieving tubal occlusion through mechanical blockade. After insertion, the microcoil induces mild mechanical tissue necrosis, leading to the release of alkaline phosphatase. This promotes local lymphocyte infiltration and vascular proliferation, thereby enhancing luminal occlusion. Furthermore, studies by Hong et al. (14) have confirmed that such mechanical tubal occlusion methods do not significantly affect ovarian function, and pregnancy outcomes are comparable to those achieved with laparoscopic proximal tubal ligation or salpingectomy (14, 15).

In terms of safety, the U.S. Food and Drug Administration (FDA) has identified potential complications associated with the Essure system, including unintended pregnancy, pain, infection, and nickel allergy (16). In contrast, among the cases treated at our center using microcoils, only two instances of microcoil misplacement and one case of tubal pregnancy were observed, with no allergic reactions reported. This relatively favorable safety profile may be attributed to the shorter follow-up period in our cohort, and we will continue monitoring patients to optimize the management of potential complications. Additionally, compared to laparoscopic procedures that may compromise ovarian blood supply (17, 18), microcoil embolization has been shown in our previous studies to significantly reduce operative time.

## 5 Limitations

This study has several limitations that should be considered when interpreting the findings. First, the relatively small sample size, particularly within the isthmic occlusion subgroup, constrains the statistical power of our analysis. This increases the risk of Type II errors and limits the generalizability of the results, including the observed clinical trend favoring bilateral over unilateral isthmic occlusion. Second, the completeness of tubal occlusion was not systematically confirmed in all cases by follow-up hysterosalpingography (HSG), which means subclinical or partial device failures cannot be entirely ruled out. Third, the absence of histological data from the occlusion sites or assessments of the endometrial microenvironment represents a significant evidence gap; such data would have provided valuable pathophysiological insights into the mechanisms behind the differential pregnancy outcomes. Future investigations with larger sample sizes, routine radiographic confirmation, and correlated histological and molecular assessments are warranted to validate and extend our observations.

## 6 Conclusion

Interstitial tubal embolization shows advantages over isthmus embolization in improving clinical pregnancy rates and reducing ectopic pregnancy risks for IVF-ET patients with tubal hydrosalpinx. Further large-scale studies are needed to confirm these benefits and optimize post-embolization IVF strategies. This technique offers a promising minimally invasive alternative for fertility preservation in such patients.
