# Correction: Efficacy of a Modified Hysteroscopic Proximal Tubal Occlusion Technique on IVF Outcomes

**DOI:** 10.3389/fmed.2025.1735047

**Published:** 2025-11-12

**Authors:** Frontiers Production Office

**Affiliations:** Frontiers Media SA, Lausanne, Switzerland

**Keywords:** hysteroscopy, *in vitro* fertilization-embryo transfer (IVF-ET), microcoil, tubal hydrosalpinx, tubal embolization

Author Tao-Tao Hu was erroneously assigned to affiliation 2. This affiliation has now been removed for author Tao-Tao Hu.

Author Yi-ling Cai was erroneously assigned to affiliation 1. This affiliation has now been removed for author Yi-ling Cai.

Author Hua-Lei Cai was erroneously assigned to affiliation 1, 2, and affiliation 3 was erroneously omitted for this author. The affiliations has been updated for author Hua-Lei Cai.

There was a mistake in Figure 3 as published. In the box “Reasons for not proceeding to embryo transfer:”, “Financial reasons: n = 1” was erroneously omitted. In the box “Reasons for not proceeding to embryo transfer:”, “Temporary personal reasons: n = 1 (for health) was erroneously omitted. The corrected Figure 3 appears below.

**Figure 3:**
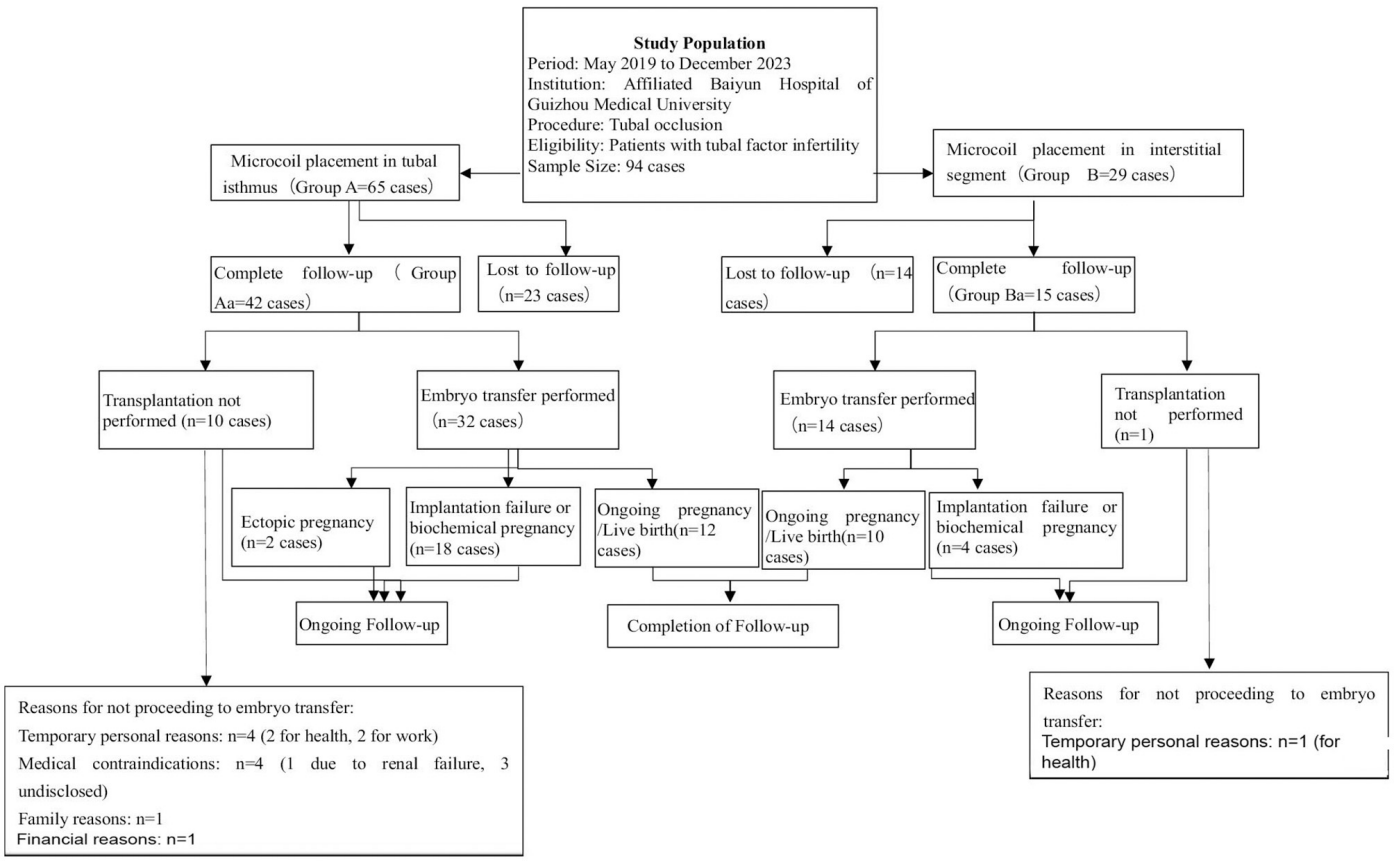
Flowchart of patient selection, tubal occlusion procedure, and follow-up outcomes in the study on tubal factor infertility.

An incorrect **Funding** statement was provided. The correct funder is the Guizhou Provincial Science and Technology Clinical Program, the correct **Funding** statement reads:

“The author(s) declare that financial support was received for the research and/or publication of this article. This study was supported by the Guizhou Provincial Science and Technology Clinical Program [Grant Number QKH-CG-LC(2024)048].”

A correction has been made to the **Results** Section, Subsection *3.4 Clinical Pregnancy Outcomes*. The words “Isthmus” and “Interstitium” were incorrectly place. The correct sentence reads: “These findings indicate that the placement of a microcoil into the interstitium (Group Ba) resulted in superior clinical outcomes compared to placement in the isthmus (Group Aa).”

The original version of this article has been updated.

