# Correction: Efficacy of a modified hysteroscopic proximal tubal occlusion technique on IVF outcomes

**DOI:** 10.3389/fmed.2025.1755575

**Published:** 2026-01-16

**Authors:** Tao-Tao Hu, Li Sun, Kai Yuan, Wen-Yu Liu, Yi-ling Cai, Hua-Lei Cai

**Affiliations:** 1 Department of Obstetrics and Gynecology, Guizhou Medical University, Guiyang, China; 2 Department of Gynecology, Bai Yun Hospital Affiliated to Guizhou Medical University, Guiyang, China; 3 Department of Obstetrics and Gynecology, Affiliated Hospital of Guizhou Medical University, Guiyang, China

**Keywords:** hysteroscopy, *in vitro* fertilization-embryo transfer (IVF-ET), microcoil, tubal hydrosalpinx, tubal embolization

The article type was erroneously given as “Review.” The correct article type is “Original Research.”

The original version of this article has been updated.

